# Galectin-8–mediated selective autophagy protects against seeded tau aggregation

**DOI:** 10.1074/jbc.M117.809293

**Published:** 2017-12-27

**Authors:** Benjamin Falcon, Jessica Noad, Harvey McMahon, Felix Randow, Michel Goedert

**Affiliations:** From the ‡ MRC Laboratory of Molecular Biology, Francis Crick Avenue, Cambridge CB2 0QH, United Kingdom,; § Department of Medicine, University of Cambridge, Cambridge CB2 0QQ, United Kingdom, and; ¶ Department of Clinical Neurosciences, University of Cambridge, Cambridge CB2 0QQ, United Kingdom

**Keywords:** autophagy, galectin, galectin-8, neurodegeneration, p62 (sequestosome 1 (SQSTM1)), protein aggregation, Tau protein (Tau), tauopathy, NDP52, seeded aggregation, prion-like

## Abstract

Assembled tau can transfer between cells and seed the aggregation of soluble tau. This process is thought to underlie the amplification and propagation of tau inclusions throughout the brain in neurodegenerative diseases, including Alzheimer's disease. An understanding of the mechanisms involved may provide strategies for limiting assembled tau propagation. Here, we sought to determine how assembled tau seeds gain access to the cytosol and whether this access triggers cellular defenses. We show that tau assemblies enter cells through clathrin-independent endocytosis and escape from damaged endomembranes into the cytosol, where they seed the aggregation of soluble tau. We also found that the danger receptor galectin-8 detects damaged endomembranes and activates autophagy through recruitment of the cargo receptor nuclear dot protein 52 (NDP52). Inhibition of galectin-8– and NDP52-dependent autophagy increased seeded tau aggregation, indicating that autophagy triggered by damaged endomembranes during the entry of assembled tau seeds protects against tau aggregation, in a manner similar to cellular defenses against cytosol-dwelling microorganisms. A second autophagy cargo receptor, p62, then targeted seeded tau aggregates. Our results reveal that by monitoring endomembrane integrity, cells reduce entry of tau seeds into the cytosol and thereby prevent seeded aggregation. The mechanisms described here may help inform the development of therapies aimed at inhibiting the propagation of protein assemblies in neurodegenerative diseases.

## Introduction

Abundant neuronal and glial inclusions composed of filamentous tau protein assemblies define a large number of sporadic human neurodegenerative diseases, collectively known as tauopathies. They include Alzheimer's disease, Pick's disease, tangle-only dementia, chronic traumatic encephalopathy, progressive supranuclear palsy, and corticobasal degeneration. Cases of frontotemporal dementia and parkinsonism linked to chromosome 17 (FTDP-17T), which are caused by mutations in the tau gene, prove that tau dysfunction is sufficient to cause neurodegeneration and dementia (1). Although the molecular species responsible for neurodegeneration are unknown, short filaments are the main species of seed-competent tau in the brains of transgenic mice expressing human P301S tau (2). Unlike Alzheimer's disease, most other tauopathies lack β-amyloid deposits. The occurrence of human tauopathies with distinct filament morphologies (3), together with experimental studies (4, 5), has led to the idea that multiple molecular conformers of aggregated tau exist. Their definition will need to be structural. As a first step, we recently presented atomic models, as determined by cryo–electron microscopy, for the cores of paired helical and straight tau filaments that were purified from the brain of an individual with Alzheimer's disease (6).

Tau inclusions originate in distinct brain regions, from where they appear to amplify and spread to anatomically connected sites (7). Experimental studies have shown that filamentous assembled tau enters cells and seeds the aggregation of soluble cytosolic tau, followed by release and uptake by neighboring cells (8, 9). Although it is known that assembled tau enters the endo-lysosomal system by endocytosis (10–12), the underlying mechanisms are only incompletely understood and it is not known how they relate to seeded aggregation. In particular, because the endo-lysosomal system does not provide direct access to the cytosol, where seeded aggregation takes place, mechanisms may exist that enable tau seeds to enter the cytosol.

The entry of microorganisms into the mammalian cytosol triggers defense mechanisms that use the autophagy cargo receptors nuclear dot protein 52 (NDP52)^3^ (also known as CALCOCO2), p62 (also known as SQSTM1), and optineurin to direct the pathogens to selective macroautophagy (hereafter referred to as autophagy) (13–15). The danger receptor galectin-8 monitors the integrity of endomembranes by binding exposed luminal glycans of ruptured vesicles, followed by binding to NDP52 and recruitment of the autophagy machinery to the vesicle remnants and their cargo (16). Cargo is then sequestered in autophagosomes, which fuse with lysosomes to mediate degradation (17). It is not known if similar defense mechanisms are active against seeded tau aggregation.

In human tauopathies, the autophagy adaptor protein p62 co-localizes with tau inclusions (18, 19). In mice transgenic for human mutant P301S tau, tau inclusions also co-localize with p62 and the autophagosome component LC3 in a ubiquitin-independent manner (20). In these mice, activation of autophagy enhances the clearance of assembled tau and reduces neurodegeneration (20, 21). Activation of autophagy also reduces seeded tau aggregation in HEK 293 cells and in induced pluripotent stem cell (iPSC)–derived neuronal cells (22, 23). However, the stages at which autophagy restricts seeded aggregation and the mechanisms involved are unknown.

To investigate if assembled tau seeds escape from the endo-lysosomal system and to study downstream effects, we looked at the roles of galectin-8, NDP52, p62, and optineurin. Here we describe how cells protect themselves against seeded tau aggregation by monitoring endomembrane integrity. Following uptake of tau seeds by clathrin-independent endocytosis, galectin-8 detected ruptured endomembranes and recruited NDP52-dependent autophagy. At later time points, seeded tau aggregates were targeted by p62.

## Results

### Clathrin-independent endocytosis of tau seeds is required for seeded tau aggregation

**Figure 1.**
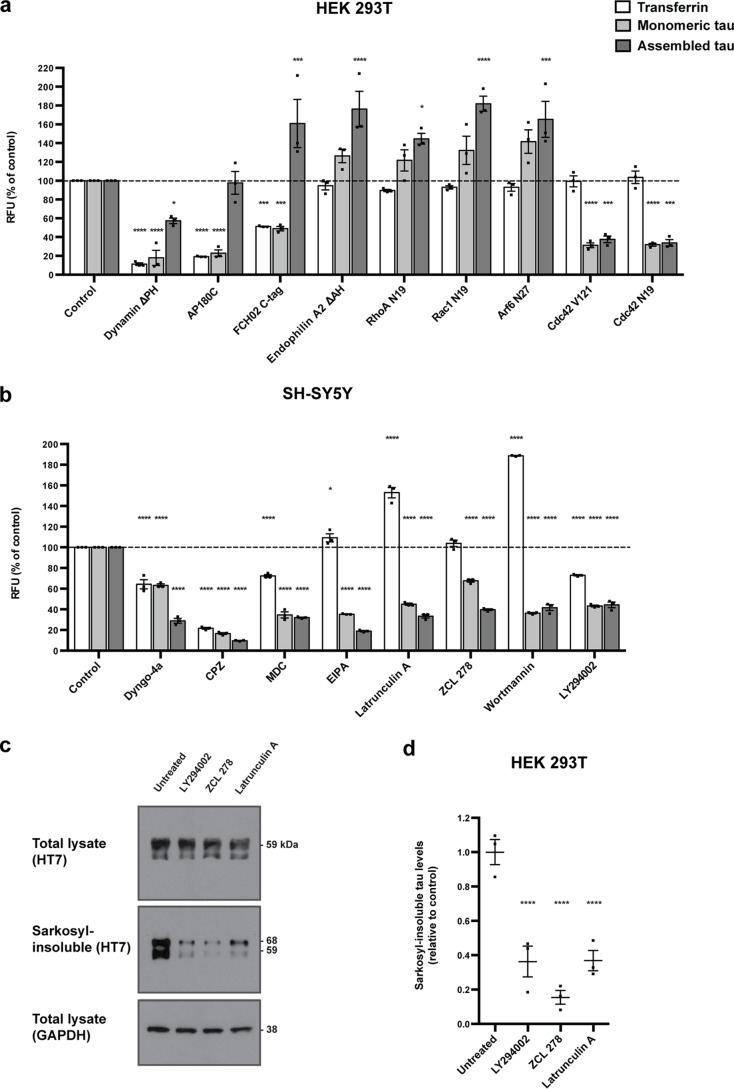
**Tau endocytosis and seeded aggregation.** *a*, flow cytometry fluorescence measurements of HEK 293T cells expressing the indicated EGFP-tagged dominant-negative mutant proteins and incubated with Alexa Fluor–labeled transferrin (*white*) or DyLight-labeled monomeric (*light gray*) or aggregated (*dark gray*) P301S tau for 30 min at 37 °C. *b*, flow cytometry fluorescence measurements of SH-SY5Y neuroblastoma cells treated with the indicated small molecule inhibitors for 30 min, followed by addition of Alexa Fluor–labeled transferrin (*white*) or DyLight-labeled monomeric (*light gray*) or aggregated (*dark gray*) P301S tau for 30 min. *a* and *b*, the results are the means ± S.E. *n* = 3; 10,000 cells/condition. *, *p* < 0.05; ***, *p* < 0.001; ****, *p* < 0.0001 (ANOVA). *c*, representative Western blots with HT7 of the total lysate and sarkosyl-insoluble fractions of HEK 293T cells expressing P301S 1N4R tau treated with aggregated P301S tau for 3 h in the presence of ZCL 278, LY294002, or latrunculin A, followed by incubation with 0.0125% trypsin and 48-h growth. GAPDH was used as a loading control. The two bands of 68 kDa and 59 kDa in the sarkosyl-insoluble fraction correspond to phosphorylated and nonphosphorylated tau, respectively. *d*, densitometric analysis of HT7 blots of the sarkosyl-insoluble fractions from *c*. The results are the means ± S.E. *n* = 3; ****, *p* < 0.0001 (ANOVA).

Monomeric and assembled full-length tau were previously shown to enter cells (12, 24). To understand the entry mechanisms, we inhibited the function of proteins involved in endocytosis through the expression of dominant-negative mutants in HEK 293T cells, or pharmacologically in SH-SY5Y cells. We then measured the uptake of fluorescently labeled monomeric and assembled recombinant P301S tau by flow cytometry (Fig. 1, *a* and *b*). Extracellular tau was removed with trypsin (12). Transferrin was used as the control.

Expression of a membrane binding–deficient, dominant-negative dynamin mutant lacking the pleckstrin homology domain (ΔPH) or treatment with the dynamin inhibitor Dyngo-4a inhibited the uptake of monomeric and assembled tau, as did expression of GTPase-inactive dominant-negative Cdc42 T17N or treatment with the Cdc42 inhibitor ZCL 278 (Fig. 1, *a* and *b*). In contrast, expression of dominant-negative Rho GTPases Arf6 T27N, RhoA T19N, and Rac1 L17N increased tau uptake, as did expression of dominant-negative endophilin lacking the amphipathic helix (ΔAH) (Fig. 1*a*). Expression of dominant-negative FCHO2 with a C-terminal red fluorescent protein (RFP) tag, or expression of dominant-negative AP180 C terminus, inhibited the uptake of monomeric tau and of transferrin, whereas assembled tau uptake was increased (Fig. 1*a*). FCHO and AP180 are required for clathrin-mediated endocytosis (25). Treatment with chlorpromazine and monodansylcadaverine inhibited the uptake of monomeric and assembled tau, in addition to inhibiting the uptake of transferrin (Fig. 1*b*). These molecules inhibit both clathrin-dependent and clathrin-independent endocytosis (26). Treatment with the PI3K inhibitors wortmannin and LY294002 inhibited tau uptake. Transferrin uptake was increased after wortmannin, as observed previously (27), and inhibited by LY294002. The actin polymerization inhibitors EIPA and latrunculin A inhibited the uptake of monomeric and assembled tau, as previously reported (11, 12), whereas transferrin uptake was increased (Fig. 1*b*). We conclude that tau monomers and tau assemblies enter cells through two distinct pathways, both of which require dynamin, actin, Cdc42, and PI3K. Endocytosis of monomeric tau is clathrin-dependent, whereas that of assembled tau is clathrin-independent.

To determine whether inhibition of assembled tau entry via actin-, Cdc42-, and PI3K-dependent pathways was sufficient to reduce seeded aggregation, 293T cells expressing P301S 1N4R tau were treated with latrunculin A, ZCL 278, and LY294002 during the uptake of assembled recombinant P301S 0N4R tau. Extracellular tau was removed with trypsin (12) and cells were grown in fresh medium without inhibitors for 48 h. Seeded P301S 1N4R tau aggregates were detected by Western blotting of the sarkosyl-insoluble fractions of cells, as described in Ref. 12. They were resolved as two bands of 68 kDa and 59 kDa, corresponding to phosphorylated and nonphosphorylated aggregated P301S 1N4R tau, respectively. P301S 0N4R tau seeds, which run at 52 kDa, were not detected. Treatment with latrunculin A, ZCL 278, and LY294002 reduced seeded tau aggregation (Fig. 1, *c* and *d*). These results define the entry pathway of the seed-competent tau species.

### Galectin-8 detects the escape of tau from the endo-lysosomal pathway to the cytosol and restricts seeded aggregation

**Figure 2.**
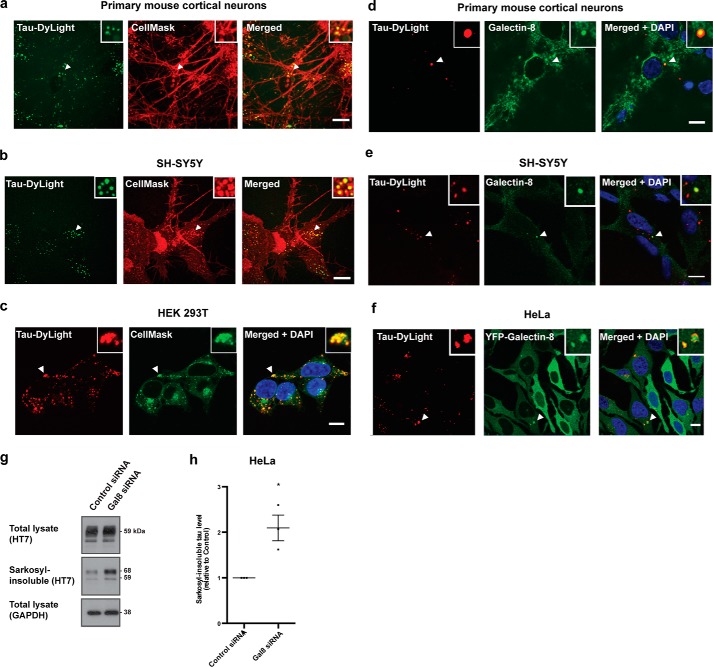
**Galectin-8 detection of tau seed escape from the endo-lysosomal system and restriction of seeded aggregation.** *a–c*, confocal images of primary mouse cortical neurons (*a*), SH-SY5Y cells (*b*), and HEK 293T cells (*c*) incubated with DyLight-labeled aggregated P301S tau (*Tau-DyLight*) and CellMask for 3 h. *d* and *e*, confocal images of primary mouse cortical neurons (*d*) and SH-SY5Y cells (*e*) treated with DyLight-labeled aggregated P301S tau (*red*) for 3 h and incubated for a further 24 h and immunostaining with anti– galectin-8 (*green*). *f*, confocal images of HeLa cells expressing YFP–galectin-8 (*green*) treated with DyLight-labeled aggregated P301S tau (*red*) for 3 h and incubated for a further 24 h. *a–f*, nuclei were visualized with DAPI (*blue*). *Arrows* indicate co-localization of tau seed–containing vesicles with CellMask/galectin-8. *Scale bars*, 10 μm. *g*, representative Western blots with HT7 of the total lysate and sarkosyl-insoluble fractions of HeLa cells expressing P301S tau and treated with galectin-8 siRNA, followed by treatment with aggregated P301S tau. GAPDH was used as a loading control. *h*, densitometric analysis of HT7 blots of the sarkosyl-insoluble fractions from *g*. The results are the means ± S.E. *n* = 3; *, *p* < 0.05 (Student's *t* test).

Assembled tau enters cells and trafficks through the endo-lysosomal system (10). We observed that fluorescently labeled assembled P301S tau seeds co-localized with the lipid membrane–binding dye CellMask in the cytoplasm by 3 h following addition to the culture medium of primary mouse cortical neurons, SH-SY5Y cells, and 293T cells, indicating that tau seeds were contained within intracellular vesicles (Fig. 2, *a–c*).

We used galectin-8 to investigate if tau seeds gained access to the cytosol from endo-lysosomal vesicles. Galectin-8 detects luminal glycans that become cytosol-exposed upon vesicle rupture (16). Immunofluorescence showed that galectin-8 antibodies detected tau seed–containing vesicles in primary mouse cortical neurons (Fig. 2*d*) and SH-SY5Y cells (Fig. 2*e*) 24 h following seed addition, with no vesicular staining in untreated cells. In SH-SY5Y cells, galectin-8 detected tau seed–containing vesicles in 6.3 ± 0.4% of cells (*n* = 3, >100 cells/coverslip). Treatment of HeLa cells expressing YFP–galectin-8 with labeled P301S tau seeds resulted in the formation of galectin-8–positive, tau seed–containing vesicles in 6.2 ± 0.2% of cells (*n* = 3, >100 cells/coverslip) (Fig. 2*f*). Galectin-8 remained diffuse in untreated cells, suggesting that damage to endo-lysosomal vesicles during tau trafficking was detected by galectin-8. Treatment of cells with filamentous assembled tau from the brains of mice transgenic for human P301S tau, which was previously shown to seed tau aggregation in cells (12), also resulted in the formation of galectin-8–positive tau seed–containing vesicles (Fig. S1*a*). In the majority of cells, galectin-8 detected damage to a single tau seed–containing vesicle, but in some cells multiple vesicles ruptured. Vesicles detected by galectin-8 were also Rab5-positive (Fig. S1*b*). This shows that during their entry into the cytosol tau seeds rupture endosomal vesicles, which is sensed by galectin-8.

Upon sensing vesicle damage caused by bacteria, galectin-8 functions as a danger receptor to restrict bacterial proliferation in the cytosol (16). To investigate if galectin-8 recruitment to tau seed–containing vesicles restricts seeded aggregation of cytosolic tau, we depleted galectin-8 in HeLa cells expressing P301S tau. Knockdown efficiency was assessed by Western blotting (Fig. S2). Cells treated with galectin-8 siRNAs displayed a 2-fold increase in seeded tau aggregation compared with control siRNA-treated cells (Fig. 2, *g* and *h*), illustrating that galectin-8 is a danger receptor that protects against seeded tau aggregation.

### Escape of tau seeds from the endo-lysosomal pathway to the cytosol limits seeded aggregation

**Figure 3.**
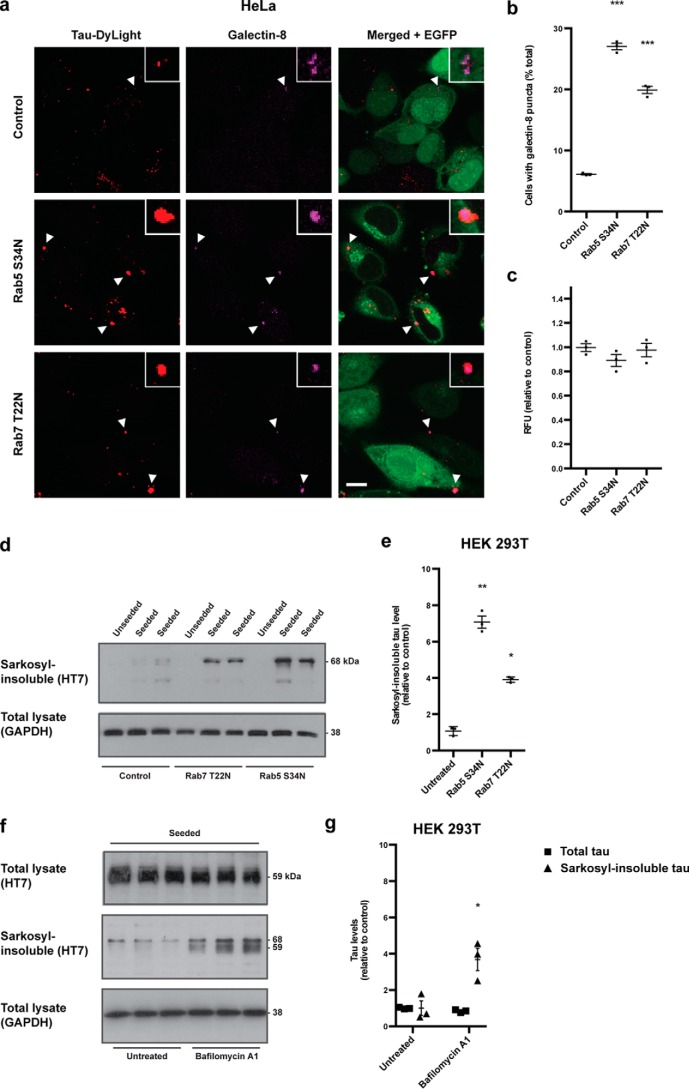
**Increased escape of tau seeds from the endo-lysosomal system facilitates seeded aggregation.** *a*, confocal images of HeLa cells expressing EGFP, Rab5 S34N-EGFP, or Rab7 T22N-EGFP (*green*) treated with DyLight-labeled aggregated P301S tau (*red*) for 3 h and incubated for a further 24 h and immunostaining with anti–galectin-8 (*magenta*). *Arrows* indicate co-localization of tau seed–containing vesicles with galectin-8. *Scale bars*, 10 μm. *b*, quantification of the percentage of cells with galectin-8–positive tau seed–containing vesicles. >100 cells counted/coverslip. *c*, flow cytometry fluorescence measurements of HeLa cells expressing EGFP, Rab5 S34N-EGFP, or Rab7 T22N-EGFP treated with DyLight-labeled aggregated P301S tau for 30 min at 37 °C. 10,000 cells/condition. *d*, representative Western blot with HT7 of the sarkosyl-insoluble fractions of 293T cells expressing P301S 1N4R tau and EGFP (*control*), Rab5 S34N-EGFP, or Rab7 T22N-EGFP treated with aggregated P301S tau for 3 h, followed by 48 h of growth. GAPDH was used as a loading control. *e*, densitometric analysis of HT7 blots of the sarkosyl-insoluble fractions from *d. f*, representative Western blot with HT7 of total lysates and sarkosyl-insoluble fractions of 293T cells expressing P301S tau treated with aggregated P301S tau for 3 h, followed by 48-h incubation with bafilomycin A1. GAPDH was used as a loading control. *g*, densitometric analysis of HT7 blots of total lysates (*squares*) and sarkosyl-insoluble (*triangles*) fractions from *f*. The results in *b*, *c*, *e*, and *g* are the means ± S.E. *n* = 3; *, *p* < 0.05; **, *p* < 0.01; ***, *p* < 0.001 (ANOVA).

To further investigate the escape of tau seeds from the endo-lysosomal system, we expressed GTPase-inactive dominant-negative Rab5 S34N and Rab7 T22N in 293T cells expressing P301S 1N4R tau. These Rab mutants arrest endo-lysosomal trafficking (28). Cells expressing Rab5 S34N and Rab7 T22N showed increased numbers of cells with galectin-8–positive tau seed–containing vesicles after 24 h compared with control cells (∼27 and ∼19% of Rab5 S34N– and Rab7 T22N–expressing cells, respectively, compared with ∼6% of control cells) (Fig. 3, *a* and *b*). In the absence of tau seeds, galectin-8 remained diffuse (Fig. S3). Uptake of assembled tau was not significantly different between cells expressing these dominant-negative proteins and control cells, indicating that increased uptake of tau seeds did not account for the increase in galectin-8–positive vesicles (Fig. 3*c*). We conclude that arresting endo-lysosomal trafficking increases the escape of tau seeds to the cytosol, possibly by extending the residence time in a susceptible exit compartment, which is detected by galectin-8.

Expression of Rab5 S34N and Rab7 T22N increased seeded tau aggregation after 48 h, by ∼7-fold and ∼4-fold, respectively, compared with empty vector controls (Fig. 3, *d* and *e*). Bafilomycin A1, which inhibits lysosomal H^+^ ATPases, also increased seeded aggregation compared with untreated cells (Fig. 3, *f* and *g*). Total tau was not changed. These results show that arresting endo-lysosomal trafficking of tau seeds increases aggregation. Lipofection of tau seeds, which increases seeded tau aggregation (29), also increased the number of cells with galectin-8–positive, tau seed–positive structures compared with cells treated with tau seeds in the absence of lipofection, with multiple galectin-8–positive structures per cell (Fig. S4). We conclude that escape of tau seeds from the endo-lysosomal pathway is limiting for seeded tau aggregation.

### NDP52-dependent and p62-dependent autophagy targets tau seeds and seeded tau aggregates, respectively

**Figure 4.**
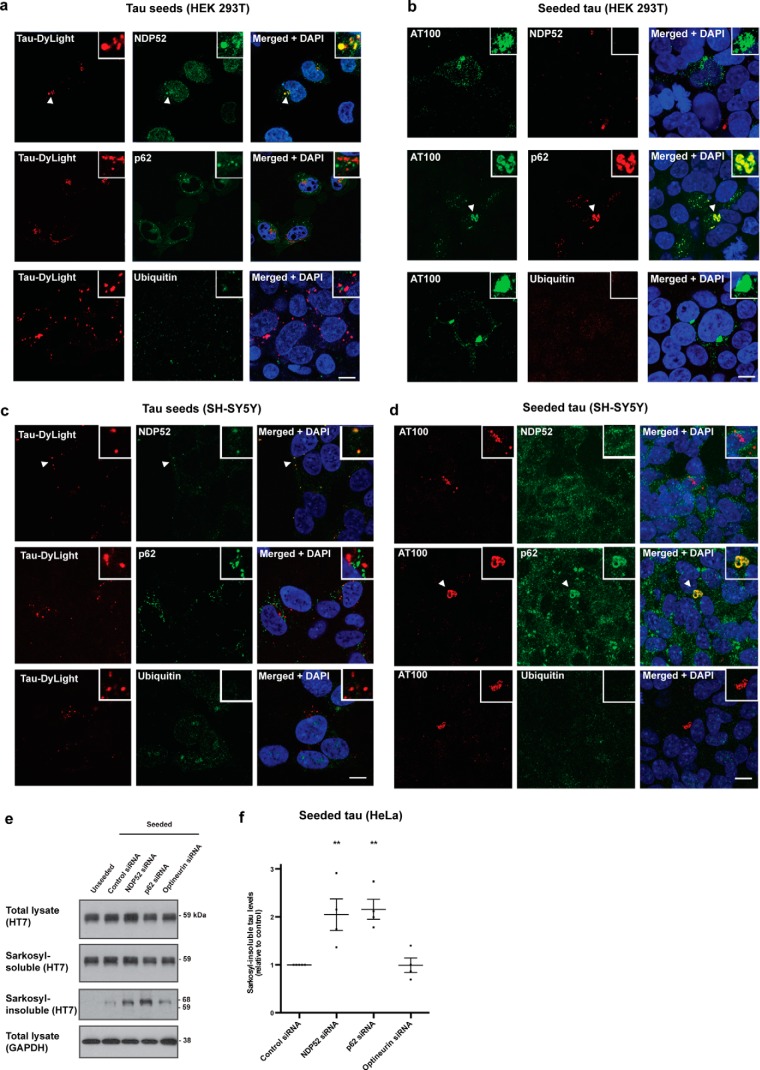
**Autophagy receptors defend against seeded tau aggregation.** *a–d*, confocal images of 293T cells (*a* and *b*) and SH-SY5Y cells (*c* and *d*) expressing P301S tau treated with DyLight-labeled aggregated P301S tau (*red*) for 3 h, followed by 24-h growth and immunostaining with anti-NDP52, anti-p62, and anti-ubiquitin (*green*) (*a* and *b*) or treated with aggregated recombinant P301S tau for 3 h, followed by 48-h growth and immunostaining with AT100 (*green*) and anti-NDP52, anti-p62, and anti-ubiquitin (*red*) (*c* and *d*). Nuclei were visualized with DAPI (*blue*). *Arrows* indicate co-localization of tau seed–containing vesicles with NDP52 and seeded tau aggregates with p62. *Scale bars*, 10 μm. *e*, representative Western blots with HT7 of the total lysate and sarkosyl-soluble and sarkosyl-insoluble fractions of HeLa cells expressing P301S tau and treated with NDP52, p62, and optineurin siRNAs, followed by treatment with aggregated P301S tau. GAPDH was used as a loading control. *f*, densitometric analysis of HT7 blots of the sarkosyl-insoluble fractions from *e*. The results are the means ± S.E. *n* = 4; **, *p* < 0.01 (ANOVA).

We asked if cells defend themselves against the entry of assembled tau seeds and seeded tau aggregation through the autophagy cargo receptors NDP52, p62, and optineurin, which were previously shown to defend against bacterial invasion (13–15). We investigated the targeting of fluorescently labeled assembled tau seeds by these autophagy cargo receptors following uptake in HeLa cells and, subsequently, the targeting of seeded tau aggregates formed after 48 h of growth. We used immunofluorescence with phosphorylation-dependent anti–tau antibody AT100 to visualize seeded tau aggregates, which are hyperphosphorylated (12), whereas tau seeds remained unphosphorylated (Fig. S5). Tau seeds were detected by NDP52, but not p62, whereas seeded tau aggregates were detected by p62, but not NDP52 (Fig. 4, *a* and *b*). Neither tau seeds nor seeded tau aggregates were detected by ubiquitin (Fig. 4, *a* and *b*). Similar results were obtained in SH-SY5Y cells (Fig. 4, *c* and *d*). Our findings suggest an orderly series of events; tau seeds are targeted by NDP52, whereas subsequently formed seeded tau aggregates are targeted by p62.

**Figure 5.**
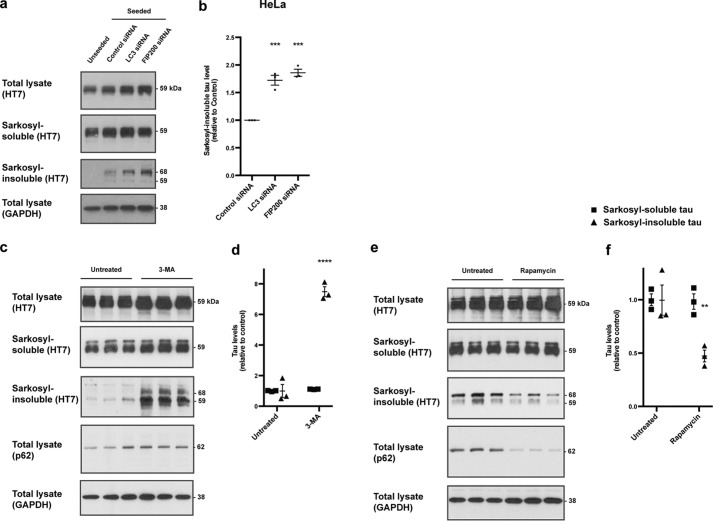
**Autophagy restricts seeded tau aggregation.** *a*, representative Western blots with HT7 of the sarkosyl-insoluble fractions of HeLa cells expressing P301S tau and treated with LC3 and FIP200 siRNAs, followed by treatment with aggregated P301S tau. GAPDH was used as a loading control. *b*, densitometric analysis of HT7 blots of the sarkosyl-insoluble fractions from *a. c*, representative Western blots with HT7 of the total lysate, sarkosyl-soluble and sarkosyl-insoluble fractions and anti-p62 of total lysates of 293T cells expressing P301S 1N4R tau treated with aggregated P301S tau for 3 h, followed by 48-h incubation with 3-methyladenine. *d*, densitometric analysis of HT7 blots of the sarkosyl-soluble (*squares*) and sarkosyl-insoluble (*triangles*) fractions from *c. e*, representative Western blots with HT7 of the total lysate, sarkosyl-soluble and sarkosyl-insoluble fractions, and anti-p62 of total lysates of 293T cells expressing P301S 1N4R tau treated with aggregated P301S tau for 3 h, followed by 48-h incubation with rapamycin. *f*, densitometric analysis of HT7 blots of the sarkosyl-soluble (*squares*) and sarkosyl-insoluble (*triangles*) fractions from *e*. In *a*, *c,* and *e*, GAPDH was used as a loading control. In *b*, *d*, and *f*, the results are the means ± S.E. *n* = 3; **, *p* < 0.01; ***, *p* < 0.001; ****, *p* < 0.0001 (ANOVA).

To investigate whether targeting by autophagy cargo receptors restricts seeded tau aggregation, we used siRNAs to deplete NDP52, p62, and optineurin in HeLa cells expressing P301S tau. Cells depleted of NDP52 and p62, but not optineurin, displayed a 2-fold increase in seeded tau aggregation compared with control siRNA-treated cells (Fig. 4, *e* and *f*). siRNA depletion of essential autophagosome components LC3C, the specific binding partner of NDP52 (30), and FIP200 also increased seeded tau aggregation (Fig. 5, *a* and *b*). Amounts of soluble tau were unchanged. Knockdown efficiency was assessed by Western blotting (Fig. S1). In addition to depletion of LC3C and FIP200, we perturbed autophagy pharmacologically following tau seed uptake. Inhibition of autophagy with 3-methyladenine (3-MA) increased seeded tau aggregation in HEK 293T cells expressing P301S tau by more than 2-fold compared with untreated cells (Fig. 5, *c* and *d*), whereas activation of autophagy by rapamycin reduced seeded tau aggregation by half (Fig. 5, *e* and *f*). Amounts of soluble tau were unchanged. Perturbation of autophagy was detected by monitoring p62 (31) (Fig. 5, *e* and *f*). Inhibition of the proteasome with MG132 and treatment with the proteasome activator rolipram had no effect on seeded tau aggregation (Fig. S6). Ubiquitinated protein load and soluble tau were increased after MG132. Taken together, our data show that seeded tau aggregation is restricted by NDP52-dependent autophagy of tau seeds and p62-dependent autophagy of seeded tau aggregates, but not by optineurin-dependent mechanisms.

### NDP52-dependent autophagy mediates the protective function of galectin-8

**Figure 6.**
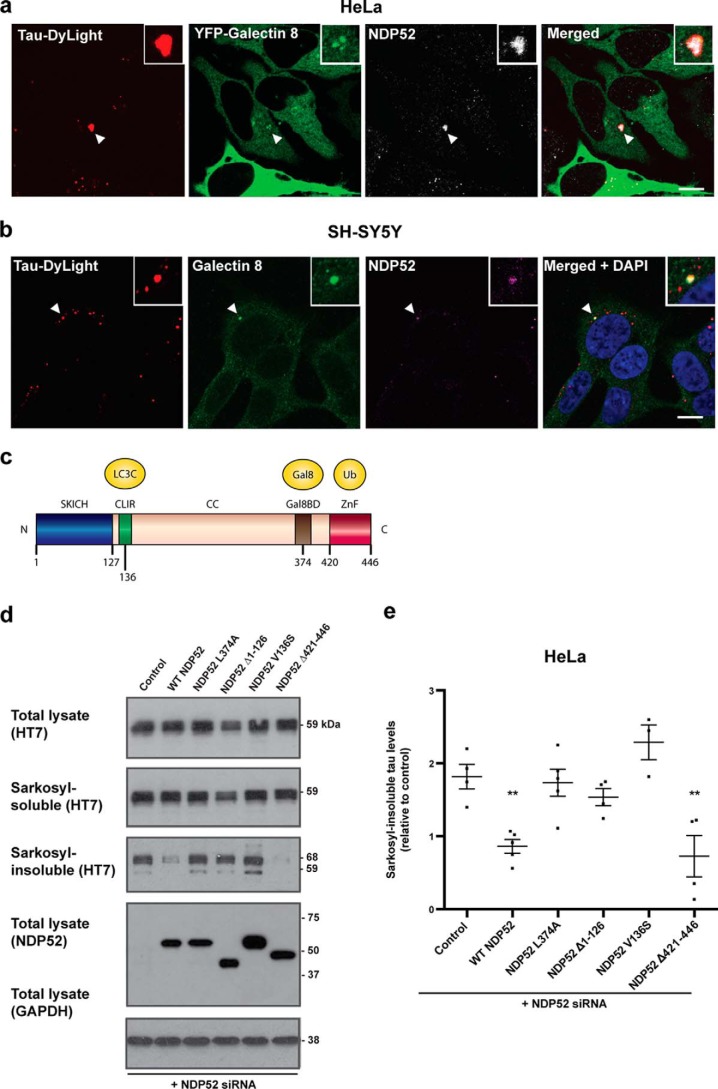
**NDP52 protects against seeded tau aggregation through galectin-8.** *a* and *b*, confocal images of HeLa cells expressing P301S tau and YFP–galectin-8 (*a*) and SH-SY5Y cells expressing P301S tau (*b*) treated with DyLight-labeled aggregated P301S tau (*red*) for 3 h, followed by 24-h growth and immunostaining with anti–galectin-8 (*green*) and anti-NDP52 (*white*/*magenta*). Nuclei were visualized with DAPI (*blue*). *Arrows* indicate co-localization of tau seed–containing vesicles, galectin-8, and NDP52. *Scale bars*, 10 μm. *c*, diagram of the known structural elements of NDP52 depicting binding partners and the point mutations used in this study. *d*, representative Western blots with HT7 and anti-NDP52 of the total lysate, sarkosyl-soluble and sarkosyl-insoluble fractions of HeLa cells expressing P301S tau and the indicated NDP52 constructs and treated with NDP52 siRNA, followed by treatment with aggregated P301S tau. GAPDH was used as a loading control. *e*, densitometric analysis of HT7 blots of sarkosyl-insoluble fractions from *d*. The results are the means ± S.E. *n* = 3; **, *p* < 0.01 (ANOVA).

NDP52-dependent autophagy mediates the restriction of bacterial proliferation by galectin-8 (16). We therefore investigated if galectin-8 protection against seeded tau aggregation (Fig. 2, *g* and *h*) was mediated by NDP52. Tau seed–containing vesicles that were detected by galectin-8 were also positive for NDP52 in HeLa cells, and SH-SY5Y cells (Fig. 6, *a* and *b*), indicating that NDP52 is recruited to ruptured tau seed–containing vesicles. NDP52 binds ubiquitinated cargo via its zinc finger (ZnF) domain and galectin-8–associated cargo via its galectin-8–binding domain (16) and recruits autophagy via its LC3C interacting region (CLIR) (30). To determine the mechanism by which NDP52 restricts seeded tau aggregation, we depleted NDP52 in HeLa cells expressing P301S tau using siRNAs and complemented with mutant NDP52 alleles (Fig. 6*c*). The expression of full-length and ubiquitin-binding–incompetent NDP52 lacking the ZnF domain (NDP52_Δ421–446_) decreased seeded aggregation, confirming the ubiquitin-independent targeting of tau seeds by NDP52 observed by immunofluorescence. By contrast, NDP52 lacking galectin-8–binding (NDP52_L374A_), LC3C-binding (NDP52_V136S_), or the SKICH domain (NDP52_Δ1–126_) failed to reduce seeded tau aggregation (Fig. 6, *d* and *e*). We conclude that NDP52 restricts seeded tau aggregation in a galectin-8–dependent, ubiquitin-independent manner by diverting tau seeds to autophagy, revealing that by monitoring the integrity of endomembranes, cells protect their cytosol against the entry of tau seeds.

## Discussion

**Figure 7.**
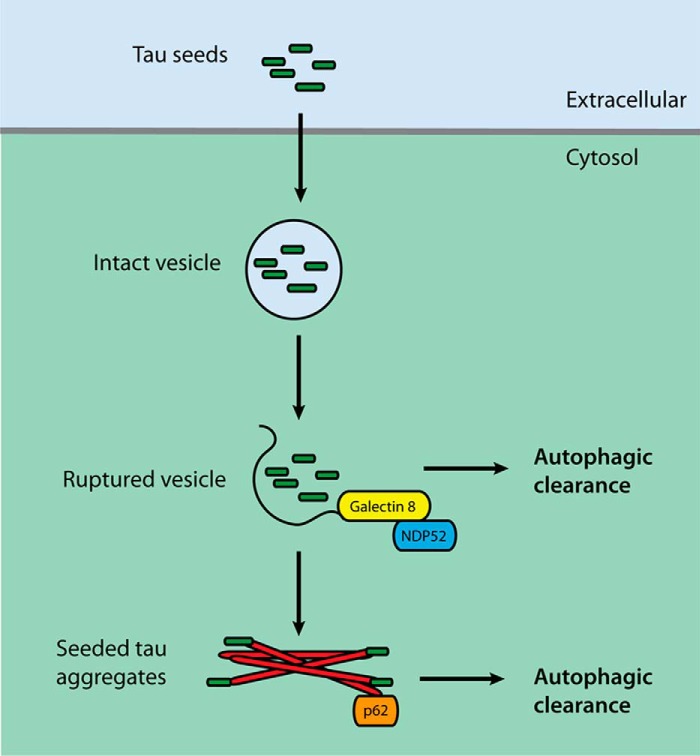
**Defensive pathways and how the factors examined in this study relate to them.**

Propagation of protein assemblies requires amplification and spread. It occurs through seeded aggregation in the cytoplasm, followed by the intercellular transfer of assemblies. Here we studied cellular defense mechanisms against assembled tau seed entry and seeded aggregation. Following clathrin-independent endocytosis, galectin-8 detected ruptured endomembranes containing tau seeds and diverted them to autophagy through binding NDP52. This restricted the formation of seeded tau aggregates, which were targeted by p62. These findings show that monitoring endomembrane integrity protects against the entry of assembled tau seeds and subsequent seeded tau aggregation in a manner similar to cell-autonomous immunity defending the cytosol against pathogen entry (Fig. 7).

We show that assembled full-length tau enters cells by clathrin-independent endocytosis, which requires actin, PI3K and Cdc42, confirming and extending previous work (10–12). This defines the uptake pathway of tau seeds, because actin-, PI3K-, and Cdc42-dependent endocytosis was required for seeded aggregation. The clathrin-independent uptake of aggregated tau also required dynamin, confirming that dynamin-dependence is not limited to clathrin-mediated endocytosis (25). It appears likely that assembled tau is heterogeneous and that only a fraction is taken up into cells and seeds aggregation (2). By contrast, monomeric tau is taken up by actin-dependent, clathrin-mediated endocytosis. In view of these different mechanisms of endocytosis, it is highly unlikely that our monomeric tau preparations contained some assembled tau, as previously suggested (32). Dependence on PI3K and Cdc42 may be linked to the requirement for actin, because these proteins regulate actin polymerization at the plasma membrane (33, 34). Alternatively, they may be required for the recycling of a receptor. Assembled tau induces endocytosis (11), suggesting that cell-surface receptors for tau may exist.

Increased tau and transferrin uptake following expression of dominant-negative mutant proteins (C-terminal–tagged FCHO, endophilin A2, RhoA T19N, Rac1 T17N, and Arf6 T27N) and small molecule inhibitors (EIPA, wortmannin, and latrunculin A), respectively, may reflect up-regulation of other endocytic pathways. Inhibition of some endocytic pathways can lead to the up-regulation of others (26).

Galectin-8 detects the rupture of endomembranes caused by tau seeds. This shows how assembled tau that is initially contained within vesicles can seed the aggregation of soluble tau in the cytosol. The mechanisms leading to the rupture of tau seed–containing endomembranes are unknown, but may involve assembled tau-dependent membrane disruption. Tau assemblies are hydrophobic and can impair the integrity of artificial membranes (35, 36). Recombinant, heparin-assembled tau also induces the formation of galectin-3 puncta in cells (36), as does the addition of cell lysates (37), which may be dependent on the presence of assembled tau in the lysates.

Escape of tau seeds into the cytosol represents a bottleneck for seeded aggregation, because most seeds were not detected by galectin-8 and because increasing escape through stalling endo-lysosomal trafficking elevated seeded assembly. Membrane barriers, in addition to galectin-8- and NDP52-dependent autophagy, therefore represent defense mechanisms against the entry of tau seeds into the cytosol. This confirms the extent to which compartmentalization of the cytosol protects cells from extracellular threats (38). Escape of tau seeds occurred mostly from endosomes, because galectin-8 detected tau seed–containing vesicles that were Rab-positive, despite its ability to detect both endosomes and lysosomes (16). Expression of dominant-negative Rab5 led to a greater increase in seeded tau aggregation than that of dominant-negative Rab7. Assembled α-synuclein may also access the cytosol through similar mechanisms, because it enters the endo-lysosomal system (39) and because galectin-3 is recruited to vesicles following their uptake (36, 40).

We have shown that cells defend themselves against seeded tau aggregation using two autophagy cargo receptors, NDP52 and p62, which act at different stages. The initial response to the escape of assembled tau seeds to the cytosol requires a galectin-8– and NDP52-dependent pathway, whereas a p62-dependent pathway targets seeded tau aggregates that form at later time points. These findings establish that galectin-8 can direct autophagy to cargo other than microorganisms and that seeded tau aggregation is primarily restricted by autophagy, rather than the proteasome. The autophagy pathways described here are ubiquitin-independent, consistent with studies in human tauopathy brains (41–43) and the central nervous system of mice transgenic for human mutant P301S tau (20). A p62-dependent pathway may be common to protein assemblies and be dependent on the PB1 domain of p62, because it is required for the ubiquitin-independent targeting of assembled SOD1 and an aggregation-prone mutant of the transcription factor STAT5A (44, 45). Unlike tau seeds assembled from recombinant protein, seeded tau aggregates were phosphorylated, indicating that tau phosphorylation is a consequence of seeded aggregation. It remains to be seen if seeded tau aggregation is influenced by other post-translational modifications.

In conclusion, we have shown that membrane barriers and mechanisms that monitor their integrity protect cells against seeded tau aggregation, in addition to colonization by microorganisms. Our findings reveal the extent to which surveying the integrity of endomembranes protects cells against seeded aggregation.

## Experimental procedures

### Cell culture

HEK 293T and HeLa cells were cultured in Dulbecco's modified Eagle's medium (DMEM) with GlutaMAX and 10% fetal calf serum (FCS). SH-SY5Y cells were cultured in minimal essential medium (MEM) and Ham's F-12 (1:1), supplemented with GlutaMAX, nonessential amino acids, and 10% FCS. Cell lines were passaged by incubation with 0.025% trypsin/EDTA for 5 min at 37 °C. They were validated and obtained from the European Collection of Authenticated Cell Cultures (ECACC). Primary mouse cortical neurons were prepared from E18.5 C57BL/6J embryos as described (46) and used for experiments 14 days after plating on poly-l-lysine–coated coverslips or dishes. Cell culture reagents were from Gibco.

### Antibodies

Anti–pThr-212/pSer-214/pThr-217 tau AT100 (MN1060) and anti-human tau HT7 (MN1000) were from Thermo Fisher; anti-GAPDH (MAB374) was from EMD Millipore; anti-Rab5 (ab18211), anti-p62 (ab91526), anti-NDP52 (ab68588), anti–galectin-8 (ab183637), and anti-ubiquitin (ab7780) were from Abcam; anti-p62 (ab610832) was from BD Biosciences; anti-LC3 (NB1000–2220) and anti-NDP52 (BO01P) were from Novus Biologicals; anti-optineurin (100000) was from Cayman; anti-FIP200 (17250–1-AP) was from Proteintech Group; anti-ubiquitin P4D1 (sc-8017) was from Santa Cruz Biotechnology; and anti–galectin-8 (AF1305) was from R&D Systems. Horseradish peroxidase (HRP)–conjugated secondary antibodies were from Bio-Rad. Alexa Fluor–conjugated secondary antibodies were from Life Sciences.

### Purification, assembly, and labeling of tau seeds

Recombinant human full-length P301S 0N4R tau was expressed and purified as described (12). Aliquots were frozen in liquid nitrogen and stored at −20 °C. Prior to an experiment, recombinant tau was centrifuged at 100,000 × *g* at 4 °C for 1 h and the supernatants used immediately. Recombinant tau (60 μm) was incubated with heparin (15 μm) (Sigma) in 30 mm MOPS, pH 7.2, with 100 μm AEBSF at 37 °C for 72 h without shaking or sonication, as described (47), to produce filamentous assemblies, as characterized in Ref. 12. Recombinant tau was covalently labeled with DyLight dyes (Thermo Fisher) using the succinimidyl ester amine reaction as described (12). Sarkosyl-insoluble filamentous assembled tau from the brains of aged mice transgenic for human P301S tau displaying a neurodegenerative phenotype (48) was extracted as described (12) and used immediately.

### Tau uptake

Tau uptake was performed as described (12). Twenty-four h before experiments, HEK 293T cells were transfected with pDEST encoding AP180 C terminus, C-terminal–tagged FCHO, dynamin ΔPH, endophilin A2 ΔAH, RhoA T19N, Cdc42 T17N, Rac1 T17N, Arf6 T27N, Rab5 S34N, and Rab7 T22N fused to enhanced GFP (EGFP) using Lipofectamine 2000 (Invitrogen). Cells were treated with 10 μg/ml transferrin conjugated to Alexa Fluor 594 (Invitrogen), followed by an acid wash (20 mm CH_3_COONa, pH 4.6, 150 mm NaCl, 1 mm CaCl_2_) for 30 s at 21 °C. CellMask (Invitrogen, 1:1000) and small molecule inhibitors were added 10 and 30 min prior to the addition of assembled tau, respectively, and were maintained in the medium throughout the experiment. The small molecule inhibitors were: 5-(*N*-ethyl-*N*-isopropyl) amiloride (EIPA, Sigma, 100 μm), latrunculin A (Sigma, 300 nm), wortmannin (Sigma, 25 nm), LY294002 (Sigma, 50 μm), Dyngo-4a (Abcam, 20 μm), ZCL278 (Tocris, 10 μm), chlorpromazine (Sigma, 28 μm), and monodansylcadaverine (Sigma, 100 μm). Fixation was with 3.6% paraformaldehyde for 10 min. 10,000 cells each from three biological replicates were analyzed by flow cytometry (Sony Eclipse or BD Biosciences LSRII).

### Seeded tau aggregation

Seeded tau aggregation was performed as described (12). Twenty-four h before experiments, HEK 293T cells were transfected with pcDNA3.1 encoding human P301S 1N4R tau and pM6P encoding galectin-8 fused to YFP using Lipofectamine 2000 (Invitrogen). SH-SY5Y cells were transfected with pcDNA3.1 encoding human P301S 1N4R tau using Lipofectamine 2000 according to Ref. 49. Cells were split into 6- or 24-well dishes and grown overnight. The medium was then replaced with assembled tau seeds (500 nm monomer equivalent) diluted in OptiMEM with GlutaMAX. After 3 h, the seed-OptiMEM mixture was replaced with growth medium and cells were grown for 48 h. Bafilomycin A1 (Sigma, 2 nm), rapamycin (Sigma, 50 nm), 3-methyladenine (Sigma, 10 mm), rolipram (Tocris, 50 μm), and MG132 (Sigma, 1 μm) were added to the growth medium during the 48-h growth period. Cells were analyzed by Western blotting or immunofluorescence staining.

### Western blotting

Cells were lysed and fractionated as described (12). The samples were resolved on 4–20% Tris-glycine gels (Novex). Following transfer, nitrocellulose membranes were blocked with 1% BSA in PBS containing 0.2% Tween 20 (PBST) for 30 min and incubated overnight at 4 °C with primary antibodies. Incubation with HRP-conjugated secondary antibodies was carried out for 1 h at 21 °C and the signal detected by enhanced chemiluminescence (GE Healthcare). Antibodies were diluted in PBST containing 1% BSA and washings carried out with PBST. The antibody dilutions were HT7 (MN1000) at 1:2500; anti-GAPDH (MAB374) at 1:3000; anti-NDP52 (ab68588) at 1:1000; anti-NDP52 (BO1P) at 1:500; anti–galectin-8 (ab183637) at 1:1000; anti-p62 (ab610832) at 1:500; anti-LC3 (NB1000–2220) at 1:500; anti-optineurin (100000) at 1:500; anti-FIP200 (17250–1-AP) at 1:1000; and P4D1 (sc-8017) at 1:1000. Densitometric analysis was performed using ImageJ (Version 1.45s, National Institutes of Health) on at least three independent experiments.

### Immunofluorescence staining

Cells were grown on poly-l-lysine–coated coverslips (BD Biosciences) and fixed with 4% paraformaldehyde for 20 min. For primary mouse cortical neurons, 2% sucrose was included in the fixation buffer. Cells were permeabilized and blocked with PBS containing 0.1% Triton X-100 and 1% BSA for 30 min. Incubation with primary antibodies was carried out overnight at 4 °C, and incubation with Alexa Fluor–conjugated secondary antibodies was carried out for 1 h at 21 °C. Antibodies were diluted in permeabilization/blocking buffer and washings carried out with PBS. The antibody dilutions were AT100 (MN1060) at 1:1000; anti-Rab5 (ab18211) at 1:200; anti-p62 (ab91526) at 1:1000; anti-NDP52 (ab68588) at 1:200; anti-ubiquitin (ab7780) at 1:1000; anti-NDP52 (B01P) at 1:100; and anti–galectin-8 (AF1305) at 1:40. DAPI (Sigma) was used to visualize cell nuclei. Cells were mounted with VECTASHIELD (Vector Laboratories). Images were acquired on a Zeiss-780 confocal microscope with a 63×, 1.4 numerical aperture oil objective. More than 100 cells per coverslip were scored in quantitative assays.

### RNAi

Stealth siRNAs (Invitrogen) and siRNA-resistant proteins were used as described (16). HeLa cells were retrovirally transduced with constructs expressing P301S 0N4R tau and siRNA-resistant NDP52 with silent mutations (underlined) (AACTCGACCTCCTCAAGCCAAAGGG). Retroviral supernatants were produced by simultaneous transfection of HEK 293T cells with vesicular stomatitis virus glycoprotein, original gag-pol, and proviral pM6P encoding the proteins using Lipofectamine 2000. Growth medium was added after 16 h and supernatants collected after 48 h. HeLa cells were transduced with 8 μg/ml polybrene (Sigma) using a range of dilutions of retroviral supernatant. After 48 h, transduced cells were selected with 5 μg/ml blasticidin. HeLa cells were transfected with siRNAs in OptiMEM using Lipofectamine 2000. After 4 h, growth medium was added and cells incubated for 3 days prior to seeding with assembled tau. The siRNAs targeted galectin-8 (GGACAAAUUCCAGGUGGCUGUAAAU), NDP52 (UUCAGUUGAAGCAGCUCUGUCUCCC), p62 (GAUCUGCGAUGGCUGCAAU), optineurin (CCACCAGCUGAAAGAAGCCUU), LC3C (GCUUGGCAAUCAGACAAGAGGAAGU), and FIP200 (GGAUUCAGAAUUGAGUGCUCUUGAA).

### Statistical analysis

All results are expressed as means ± S.E., and *n* values refer to the number of independent experiments. Statistical comparisons between groups were performed using the two-tailed Student's *t* test or by analysis of variance (ANOVA), as detailed in the figure legends. Dunnett's test was used to control for multiple comparisons. GraphPad Prism 7 software was used to perform analyses.

## Author contributions

All authors contributed to designing the experiments. B.F. and J.N. conducted the experiments and data analyses. B.F., J. N., H. M., F.R., and M.G. wrote the manuscript.

## Supplementary Material

Supporting Information

## Footnotes

3 The abbreviations used are:

NDP52: nuclear dot protein 52
PH: pleckstrin homology domain
EIPA: 5-(*N*-ethyl-*N*-isopropyl) amiloride
AEBSF: 4-(2-aminoethyl)benzenesulfonyl fluoride hydrochloride
EGFP: enhanced GFP.
