# Supplementary material for: Galectin-8–mediated selective autophagy protects against seeded tau aggregation
Source: J Biol Chem. 2017 Dec 27;293(7):2438–51. doi: 10.1074/jbc.M117.809293 (PMC5818177; doi:10.1074/jbc.M117.809293)
Supplement: Supporting Information [file supp_293_7_2438__index.html]

Galectin-8–mediated selective autophagy protects against seeded tau aggregation — Galectin-8–mediated autophagy and seeded tau aggregation — Supporting Information 

# Galectin-8–mediated selective autophagy protects against seeded tau aggregation

## Supporting Information

- Supplemental data (.pdf, 790 KB) - Supplemental data
