# Supplementary material for: Galectin-8–mediated selective autophagy protects against seeded tau aggregation
Source: J Biol Chem. 2017 Dec 27;293(7):2438–51. doi: 10.1074/jbc.M117.809293 (PMC5818177; doi:10.1074/jbc.M117.809293)
Supplement: Supporting Information [file 10.1074_M117.809293_jbc.M117.809293-1.pdf]

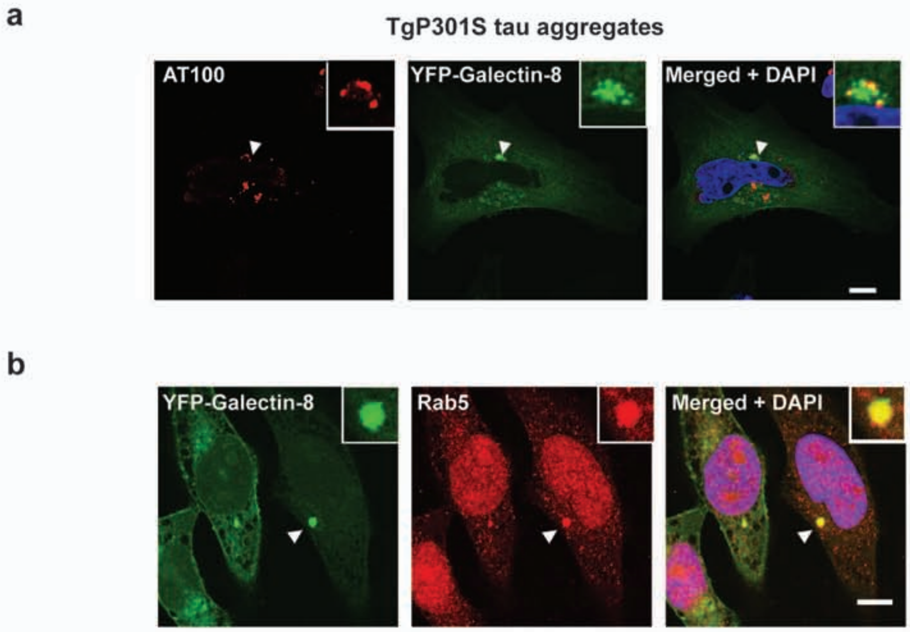

Figure S1: Galectin-8 detection of native tau seed escape from the endo-lysosomal system
a. Confocal images of HeLa cells expressing YFP-galectin-8 (green) treated with aggregated tau from TgP301S tau mice for 3 h and incubated for a further 24 h and immunostaining with AT100 (red). b. Confocal images of HeLa cells expressing YFP-galectin-8 (green) treated with aggregated P301S tau for 3 h, followed by 24 h growth and immunostaining with anti-Rab5 (red). Arrows indicate co-localisation of tau seed-containing vesicles/ Rab5-positive vesicles with galectin-8. Scale bars, 10 μm.

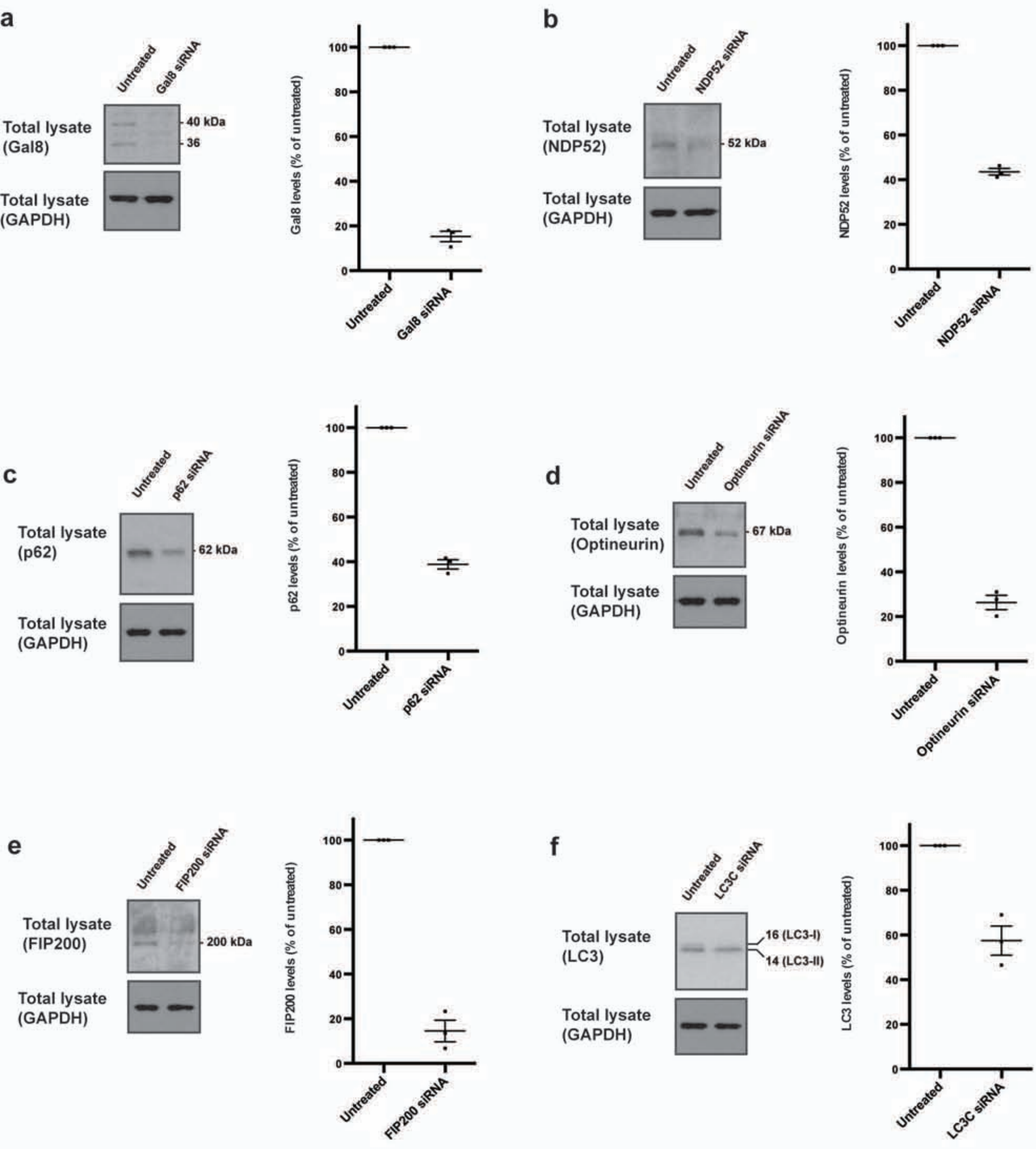

Figure S2: Knock-down efficiency of siRNAs

Representative Western blots with the indicated primary antibodies of the total lysate of HeLa cells expressing P301S tau and treated with (a.) Galectin-8, (b.) NDP52, (c.) p62, (d.) Optineurin, (e.) FIP200 and (f.) LC3 siRNAs. GAPDH was used as a loading control. Corresponding densitometric analysis is shown. The results are the means ± SEM. n=3.

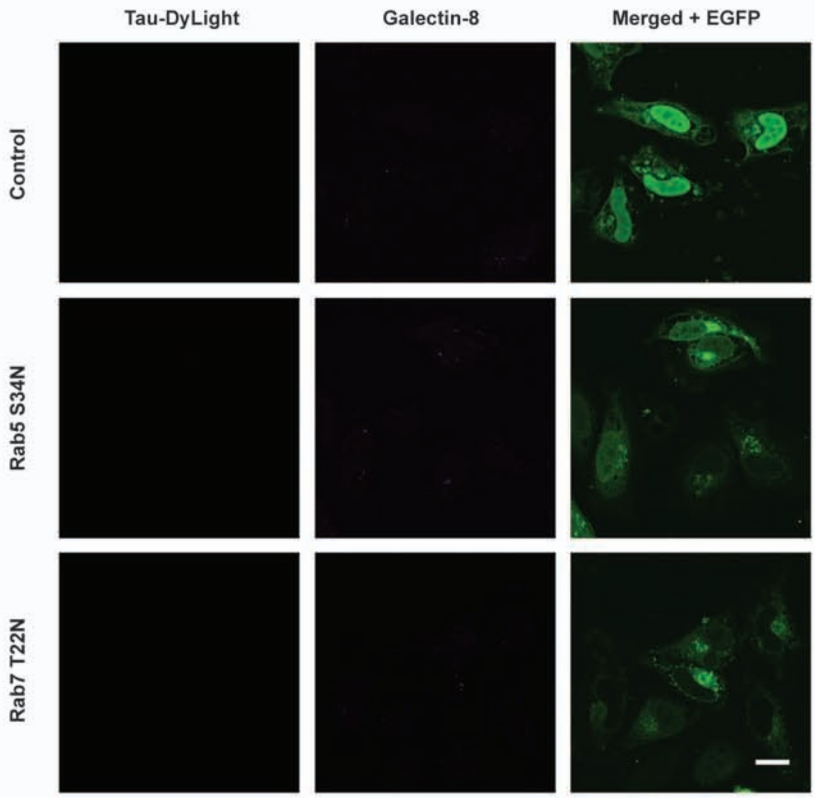

Figure S3: Lack of vesicular galectin-8 staining in cells not treated with tau seeds
Confocal images of HeLa cells expressing EGFP, Rab5 S34N-EGFP or Rab7 T22N-EGFP (green), without the addition of tau seeds, grown for 24 h and immunostaining with anti-galectin8 (magenta). Scale bar, 10 µm.

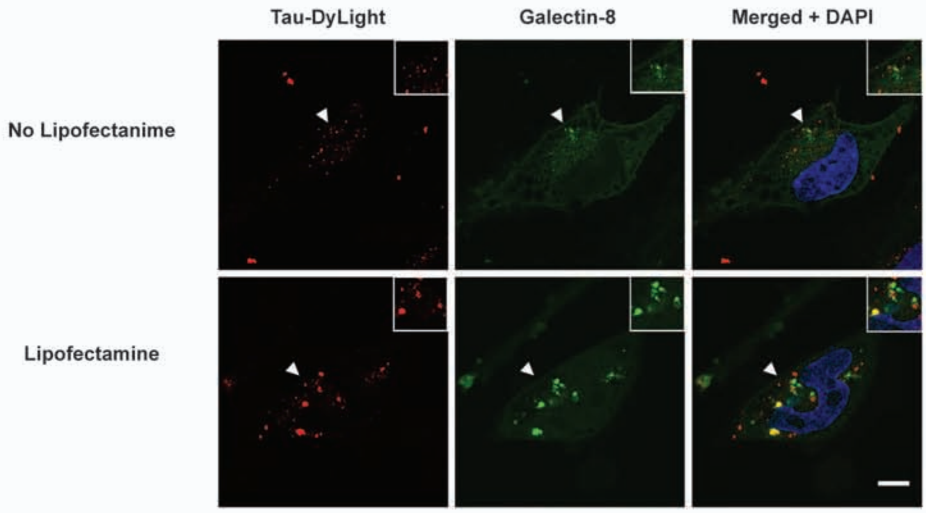

Figure S4: Lipofection increases the detection of tau seeds by galectin-8
Confocal images of HeLa cells treated with DyLight-labeled aggregated P301S tau (red) for 3 h (with and without pre-incubation with Lipofectamine 2000) and incubated for a further 24 h and immunostaining with anti-galectin8 (green). Nuclei were visualized with DAPI (blue). Arrows indicate co-localisation. Scale bar, 10 μm.

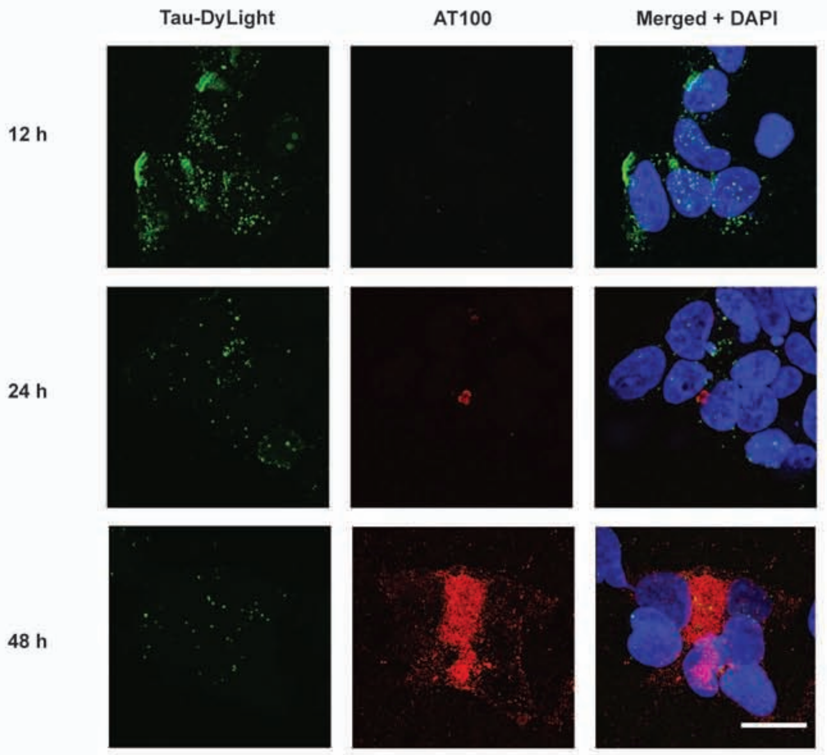

Figure S5: Tau seeds are not detected by the antibody AT100
Confocal images of HeLa cells treated with DyLight-labeled aggregated P301S tau (green) for 3 h, followed by incubation for the indicated number of hours and immunostaining with AT100 (red). Nuclei were visualized with DAPI (blue). Scale bar, 20 µm.

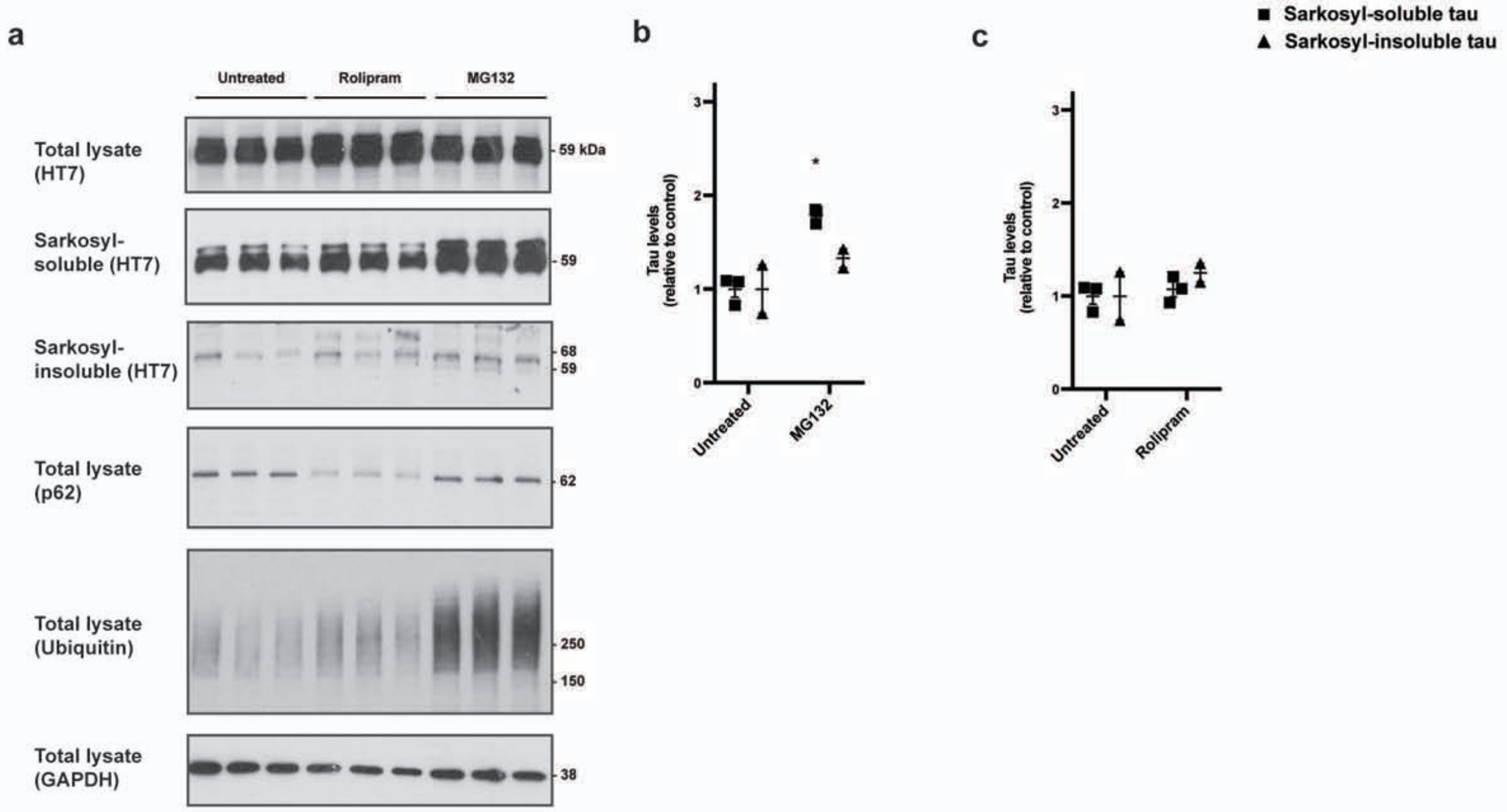

Figure S6: Effects of inhibition and activation of the proteasome on seeded tau aggregation

a. Representative Western blots with HT7 of total lysates, sarkosyl-soluble and sarkosyl-insoluble fractions, and anti-p62 and anti-ubiquitin of total lysates of 293T cells expressing P301S 1N4R tau treated with aggregated P301S tau for 3 h, followed by 48 h incubation with rolipram or MG132. GAPDH was used as a loading control. b and c. Densitometric analysis of HT7 blots of the sarkosyl-soluble (squares) and sarkosyl-insoluble (triangles) fractions of MG132-treated cells (b) and rolipram-treated cells (c) from a. The results are the means ± SEM. n=3. * $p<0.05$ (ANOVA).
